# IgG4 Characteristics and Functions in Cancer Immunity

**DOI:** 10.1007/s11882-015-0580-7

**Published:** 2016-01-07

**Authors:** Silvia Crescioli, Isabel Correa, Panagiotis Karagiannis, Anna M. Davies, Brian J. Sutton, Frank O. Nestle, Sophia N. Karagiannis

**Affiliations:** St. John’s Institute of Dermatology, Division of Genetics and Molecular Medicine; Faculty of Life Sciences and Medicine, King’s College London, London, UK; NIHR Biomedical Research Centre at Guy’s and St. Thomas’s Hospitals and King’s College London, King’s College London, London, UK; Randall Division of Cell and Molecular Biophysics, Faculty of Life Sciences and Medicine, King’s College London, London, UK; Medical Research Council & Asthma UK Centre in Allergic Mechanisms of Asthma, London, UK; St. John’s Institute of Dermatology, Division of Genetics and Molecular Medicine, Kings’ College London and NIHR Biomedical Research Centre at Guy’s and St. Thomas’s Hospitals and King’s College London, Guy’s Hospital, Tower Wing, 9th Floor, London, SE1 9RT UK

**Keywords:** IgG4, Cancer, Immune escape, Antibodies, Effector functions, Immunotherapy

## Abstract

IgG4 is the least abundant subclass of IgG in normal human serum, but elevated IgG4 levels are triggered in response to a chronic antigenic stimulus and inflammation. Since the immune system is exposed to tumor-associated antigens over a relatively long period of time, and tumors notoriously promote inflammation, it is unsurprising that IgG4 has been implicated in certain tumor types. Despite differing from other IgG subclasses by only a few amino acids, IgG4 possesses unique structural characteristics that may be responsible for its poor effector function potency and immunomodulatory properties. We describe the unique attributes of IgG4 that may be responsible for these regulatory functions, particularly in the cancer context. We discuss the inflammatory conditions in tumors that support IgG4, the emerging and proposed mechanisms by which IgG4 may contribute to tumor-associated escape from immune surveillance and implications for cancer immunotherapy.

## Introduction

### IgG Structure

The four human IgG subclasses were discovered in the 1960s and named according to their time of discovery and order of their relative abundance in human serum (approximately IgG1, 61 %; IgG2, 32 %; IgG3, 4 %; and IgG4, 3 %) [1, 2•]. IgGs are heterotetrameric glycoproteins composed by two identical light chains and two identical heavy chains. Each chain comprises a series immunoglobulin domains [3]. Heavy chains (γ1, γ2, γ3, or γ4, ∼50 kDa) are composed of an N-terminal variable domain (V_H_) followed by three constant domains (C_H_1, C_H_2, and C_H_3). Light chains (κ or λ, ∼25 kDa) are composed of an N-terminal variable domain (V_L_) and a constant domain (C_L_).

**Fig. 1:**
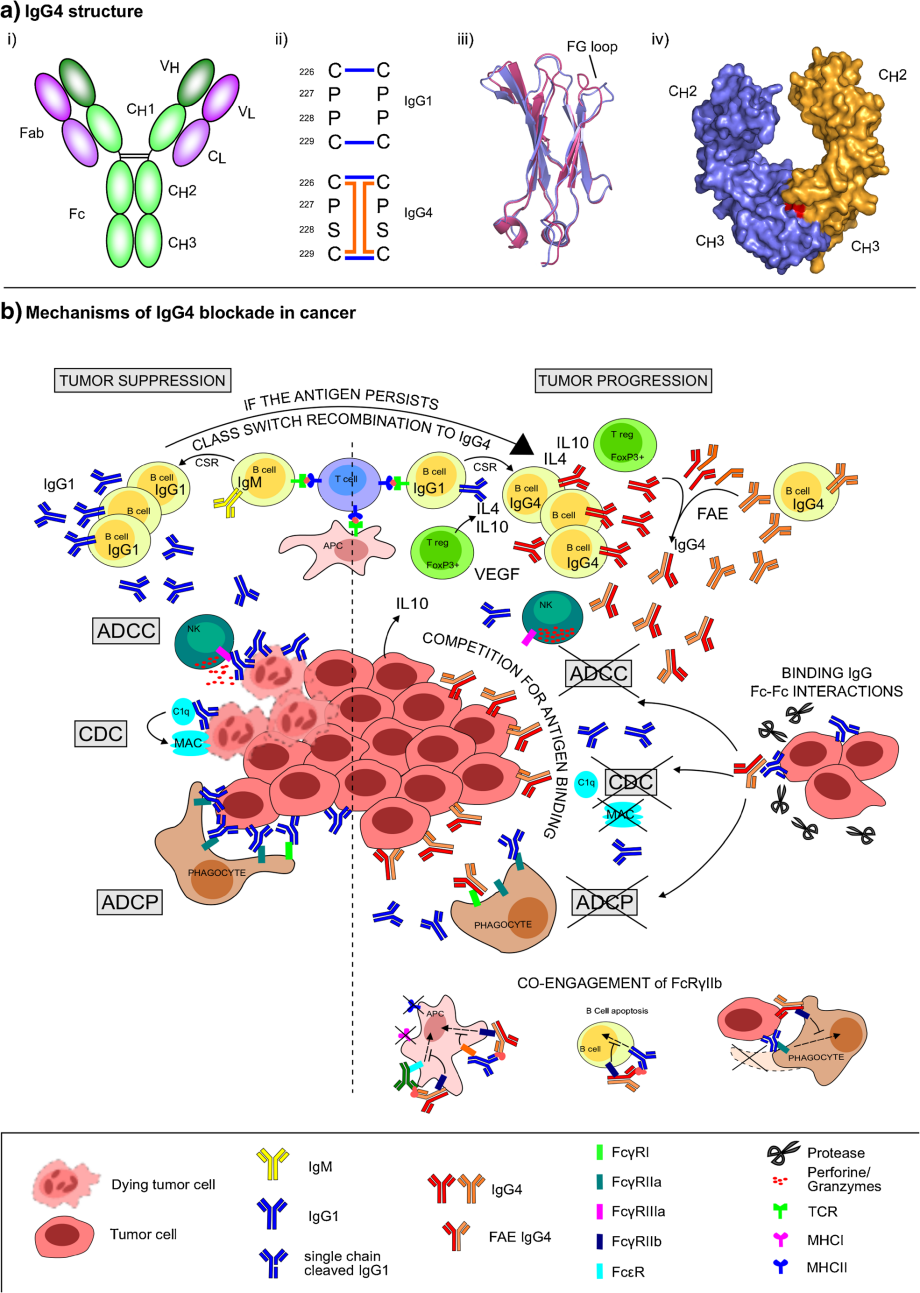
Structural and functional features of IgG4. **a** IgG4 structure: (*i*) IgG architecture. The Fab comprises V_H_, V_L_, C_H_1, and C_L_ domains. The Fc region comprises C_H_2 and C_H_3 domains, and the hinge connects the Fab to the Fc. In IgG1, two disulfide bonds (*black lines*) covalently link the two heavy chains. (*ii*) The core hinge. In IgG1, residues 226–229 from the core hinge are CPPC. Inter-heavy chain disulfide bonds form between Cys226 and Cys229 (*blue lines*). Residues 226–229 are CPSC. In addition to the hinge disulfide bond pattern in IgG1, intra-heavy chain disulfide bonds can form between Cys226 and Cys229 (*orange lines*). (*iii*) Structure of the IgG C_H_2 domain. The IgG1 and IgG4 C_H_2 domains are *colored in pink* and *blue*, respectively. While the overall fold of the C_H_2 domains is similar, in IgG4, the C_H_2 domain FG loop adopts a unique conformation. (*iv*) Crystal structure of the IgG4-Fc region. The two heavy chains are *colored in blue* and *orange*. Arg409, positioned at the interface of the C_H_3-C_H_3 domain dimer, is *colored in red*. **b** Proposed mechanisms of IgG4 blockade in cancer. Tumor-associated humoral immunity could promote tumor suppression or progression (*left* and *right* of *dotted line*, respectively). Rapid production of anti-tumor IgG1 can eliminate antigen-expressing tumor cells through CDC, ADCC, and ADCP. Chronic antigen persistence along with a Th2-biased cytokine milieu (IL-4, IL-10, VEGF) expressed by resident Tregs and tumor cells can support sequential CSR of B cells to IgG4. IgG4 might thus be more affinity matured than clonally related IgG1 and may compete with IgG1 for binding tumor antigens. IgG4 could also undergo Fab-arm exchange with other IgG4s, resulting in functionally monovalent antibodies or antibodies with increased avidity. Inability of IgG4 to fix complement and to bind activating FcγRs on immune effector cells may result in blockade of antibody-mediated CDC, ADCC, and ADCP. Alternatively, binding of IgG4 to the inhibitory FcγRIIb with higher affinity than other IgG subclasses could form ICs together with other antibodies, such as IgG1, co-engaging FcγRIIb and activating FcγRs, dampening FcγR-mediated functions. Also, in the proteolytic conditions of tumor microenvironments (e.g., MMP), IgG1 bound to tumors could be cleaved on one heavy chain, causing partial dissociation and facilitating IgG4-Fc binding. This could interfere with IgG1-mediated effector functions or trigger intracellular uptake and clearance of the target from tumor cell surfaces

Light chain V_L_ and C_L_ domains are paired with heavy chain V_H_ and C_H_1 domains, respectively, to form the fragment antigen-binding (Fab) arms. The complementarity-determining regions (CDRs) from the V_H_ and V_L_ domains form the antigen-binding site. Between the C_H_1 and C_H_2 domains lies the flexible hinge, which connects the Fab to the fragment crystallizable (Fc) region, comprising C_H_2 and C_H_3 domains. The Fc region is involved in antibody effector functions, engaging complement or FcγRs to trigger activation of immune effector cells. The two light/heavy chain (HL) pairs are joined to form the whole antibody through covalent and non-covalent interactions between the heavy chains (Fig. 1a).

IgG4 contains unique structural features in the hinge, C_H_2 and C_H_3 domains, that are thought to be responsible for its structural properties, binding characteristics and reduced effector function, compared to other subclasses.

### IgG Production by B Cells and Class Switching

Proliferating B cells undergo class switch recombination (CSR), enabling them to produce antibodies with the same antigen specificity, but different isotypes (and subclasses), with different Fc regions, and consequently different effector functions. This affords the flexibility to develop a variety of immune responses against the same target, immediately upon antigenic stimulation or in a temporal manner. CSR entails intra-chromosomal DNA recombination between the region located downstream the Variable Diversity Joining segments (VDJ) region (that encode V_H_) and the region upstream (a specific C_H_ gene). The process is triggered by the enzyme activation-induced cytidine deaminase (AID). CSR occurs when B cells are stimulated by T cells in the germinal centers, and the switch toward one specific class is dependent on the cytokine milieu in the B cell microenvironment [4]. Isotype switching to both IgG4 and IgE is known to be promoted by Th2-type cytokines (IL-4, IL-13) and by B and T cell interaction through CD40:CD40-ligand [5, 6]. While in the so-called Th2-biased response, biased expression of the cytokines IL-10, VEGF, [6, 7], IL-12 [8], and IL-21 [8, 9] have been reported to skew class switching toward IgG4.

### Binding Characteristics of IgG Subclasses for Fcγ Receptors

Human FcγRs are expressed in different immune cells subsets (Table 1) [10–12] and can be divided into two groups depending on their ability to bind monomeric IgG (high-affinity receptors) or exclusively IgG immune complexes (IC) (low-affinity receptors) [13]. There are three families of FcγRs. FcγRI (CD64) is the only high-affinity receptor. The other two families comprise the low-affinity receptors, FcγRII (CD32) and FcγRIII (CD16). The FcγRII family comprises FcγRIIa, FcγRIIb ,and FcγRIIc, while the FcγRIII family comprises FcγRIIIa and FcγRIIIb [10]. FcγRs are functionally divided into activating and inhibitory receptors. All the receptors have activating properties except FcγRIIb which is inhibitory, and FcγRIIIb whose function is uncertain. The affinity of IgG4 for FcγRI is of the same order of magnitude as IgG1 and IgG3, while IgG4 binds to the other activating receptors with lower affinity. The affinity of IgG4 for the inhibitory FcγRIIb is similar or even higher than that of the other subclasses (Table 1) [12].

**Table 1:** Structural and functional properties of IgG subclasses

IgG Attributes	FcγR-expressing human immune cells^a^	Antibody subclass			
		IgG1	IgG2	IgG3	IgG4
Molecular mass (kDa)		146	146	170	146
Hinge length		15	12	62^b^	12
Inter-heavy chain disulfide bonds (numbers in the hinge/molecule)		2	4^b^	11^b^	2
Serum half-life (days)		21	21	7	21
Relative serum abundance (% of total IgG)		60	32	4	4
C1q binding		++	+	+++	−
Affinities (scores^c^ and K_A_ values, M^−1 d^) for FcγRs					
FcγRI (activating)	Constitutive expression: monocytes, macrophages, dendritic cells (DCs) Inducible expression: mast cells, neutrophils	+++ K_A_ 6.5 × 10^7^ M^−1^	−	++++ K_A_ 6.1 × 10^7^ M^−1^	++ K_A_ 3.4 × 10^7^ M^−1^
FcγRIIa (activating)	DCs, monocytes, macrophages, neutrophils	+++ K_A_ 3.5 (R131) − 5.2 (H131) × 10^6^ M^−1^	++ K_A_ 0.1 (R131) − 0.45 (H131) × 10^6^ M^−1 b^	++++ K_A_ 0.89 (H131) − 0.91 (R131) × 10^6^ M^−1 b^	++ K_A_ 0.17 (H131) − 0.21 (R131) × 10^6^ M^−1^
FcγRIIb (inhibitory)	B cells, macrophages, mast cells, basophils, DCs (monocytes and neutrophils mainly in spleen and lymph nodes rather than in the blood)	+ K_A_ 0.12 × 10^6^ M^−1^	− K_A_ 0.02 × 10^6^ M^−1^	++ K_A_ 0.17 × 10^6^ M^−1^	++ K_A_ 0.20 × 10^6^ M^−1^
FcγRIIc (activating)	Natural killer (NK) cells (20 % of the human population), monocytes, neutrophils	+ K_A_ 0.12 × 10^6^ M^−1^	− K_A_ 0.02 × 10^6^ M^−1^	++ K_A_ 0.17 × 10^6^ M^−1^	++ K_A_ 0.20 × 10^6^ M^−1^
FcγRIIIa (activating)	NK cells, monocytes, macrophages	++ K_A_ 1.2 (F158) – 2 (V158) × 10^6^ M^−1^	−/+ K_A_ 0.03 (F158) – 0.07 (V158) × 10^6^ M^−1 b^	++++ K_A_ 7.7 (F158) – 9.8 (V158) × 10^6^ M^−1 b^	− K_A_ 0.20 (F158) – 0.25 (V158) × 10^6^ M^−1^
FcγRIIIb (unknown function)	Neutrophils, basophils	+++ K_A_ 0.2 × 10^6^ M^−1^	–	++++ K_A_ 1.1 × 10^6^ M^−1^	–

^a^FcγR-expressing human immune cells adapted from Bruhns et al. (2012) [10] and Nimmerjahn et al. (2015) [11]

^b^Values vary depending on antibody allotypes

^c^Affinity values are based on IgG immune complex (IC) binding to FcγR-transfected cells, adapted from Bruhns et al. (2009) [12]

^d^K_A_ affinity values were determined by surface plasmon resonance (SPR) analysis of binding of monovalent IgGs to immobilized FcγR recombinant ectodomains, adapted from Bruhns et al. (2009) [12]

The poor affinity of IgG4 for the activating receptors, except FcγRI, results in an impaired ability to engage immune effector cells compared to IgG1, its characteristics and its relevance in cancer are discussed in this review [14].

## Characteristics that Give IgG4 Distinct Binding and Functional Properties

Although the constant heavy chain regions of different IgG subclasses share over 95 % sequence homology, their structures and effector functions differ. IgG4 in particular has unique characteristics that may be responsible for its anti-inflammatory properties and less potent effector function compared with IgG1 and IgG3 [15]. Throughout this review, amino acids are indicated using the single letter code and their position is indicated according to the EU-index numbering [16].

### The Hinge and C_H_2 Domains Are Responsible for Poor Binding to C1q and FcγRs

The structure of IgG4 combines a short hinge and low Fab-arm flexibility [17]. Since the composition and length of the hinge affects the conformation and the flexibility of the Fab arms relative to each other, and relative to the Fc region, the orientation of the IgG4 Fab arms may partly shield the C1q and FcγR binding sites on the C_H_2 domain [18, 19].

Together with the lower hinge region, the FG loop (loop between strand F and strand G) from the C_H_2 domain is also known to be crucial for IgG binding to FcγRs [20, 21] and to C1q [22, 23]. Recent high resolution crystal structures of human IgG4-Fc showed that the FG loop in the IgG4 C_H_2 domain can adopt a unique conformation that disrupts the C1q and FcγR binding sites [24••] (Fig. 1a).

These features combined could be responsible for the poor ability of IgG4 to engage C1q and FcγRs and could result in low capacity to trigger effector functions.

### Fab-arm Exchange Affects Bivalency

A distinct property of IgG4 is its ability to undergo a process termed Fab-arm exchange, in which “half molecules,” each comprising one heavy and one light chain (HL), exchange with IgG4 antibodies of different specificities, resulting in the formation of bi-specific antibodies [15]. FAE has been demonstrated to occur in vivo [25, 26].

Structurally, the two half HL molecules are held together by covalent (hinge disulfide bonds) and non-covalent (C_H_3-C_H_3) interactions. Two features, namely the core hinge sequence and residue 409 at the C_H_3-C_H_3 domain interface, which both weaken the interactions between the two half HL molecules, are responsible for the ability of IgG4 to undergo FAE (Fig. 1a).

In IgG1, which does not undergo FAE, residues 226–229 in the core hinge have a Cysteine-Proline-Proline-Cysteine (CPPC) sequence motif. On the other hand, the IgG4 core hinge has a Cysteine-Proline-Serine-Cysteine (CPSC) motif. The IgG4 core hinge is believed to be more flexible, promoting the formation of intra-heavy chain disulfide bonds, Thus, two hinge isomers are possible, one with the typical inter-heavy chain disulfide bonds (covalently linked half molecules), and the other with intra-heavy chain disulfide bonds (non-covalently linked half molecules) [26, 27] (Fig. 1a). The S228P mutation, which renders the IgG4 core hinge more IgG1-like, abolishes the formation of intra-chain disulfide bond isomers and abrogates FAE in vitro and in vivo [26, 27].

Additionally, the IgG4 C_H_3 domain differs from the IgG1 C_H_3 domain in that lysine 409 in IgG1 is substituted for arginine in IgG4 (Fig. 1a). Residue 409 is located at the interface between the C_H_3 domain dimer. Arg409 disrupts the inter-domain network of water-mediated hydrogen bonds that is conserved in IgG1 and weakens the non-covalent interaction between the C_H_3 domains [28, 29••].

A direct consequence of Fab-arm exchange is the production of IgG4 antibodies with random dual specificity, unable to cross-link identical antigens and therefore perhaps unable to form large IC against a specific target. For this reason, such IgG4 molecules may be defined as functionally monovalent [30, 31].

A different interpretation of FAE may be that bispecificity, the ability to bind two antigens, is a property that might increase the avidity of the antibody if these antigens are proximally located on target cells [32]. If bi-specific IgG4 could crosslink two different antigens, for instance on the surface of a tumor cell, the effects of these interactions are far from clear, and these interactions might promote or restrict target cell signaling and growth.

### A Temporal Model for Isotype Switching: Potential Implications for Higher Antigen Affinities for IgG4

An analysis of almost 1000 VDJ sequences extracted from B cells of individuals living in an area of endemic parasitism showed a positive correlation between the number of mutations somatic hypermutation (SHM) and the distance of the specific Cγ gene from the VDJ region in the heavy chain locus. The Cγ4 locus is the last of the IgG subclasses on chromosome 14 [33••]. The authors proposed a temporal model, where CSR occurred sequentially from IgM to IgG3 then to IgG1, to IgG2, and finally to IgG4, with the implication that class-switched IgG4 B cells would be the last to exit from germinal centers. Since AID is involved both in CSR and SHM, B cells would also accumulate mutations in the VDJ region, leading to the production of antibodies with higher affinity (affinity matured) [32]. Most activating immune responses induce the rapid production of IgG3 and IgG1, but not of the other isotypes, possibly due to the rapid elimination of the antigen-expressing targets. However, if the antigen persists, B cells continue to be activated in the germinal centers and undergo sequential CSR to IgG2 and IgG4.

Notably, the Cγ position on heavy chain locus and therefore the extent of SHM, also positively correlate with antibody effector functions (lower ability to activate complement and FcγRs for IgG2 and IgG4). This could be interpreted as a mechanism of self-regulation by the immune system to avoid self-damage in response to a chronic antigenic stimulus. According to the CSR temporal model, IgG4 has higher affinity for antigen compared to IgG1 or IgG3, while it also has poor capacity to activate complement and FcγRs. Evidence to support this model exists in the context of allergy [34] and of endemic parasitism [32, 33••].

In cancer, high-affinity tumor-specific IgG4 could compete with IgG1 for the binding to tumor-associated antigens. Since IgG4 has lower ability to trigger effector functions compared to IgG1, this competition results in reduced antibody-mediated effector functions and escape from the immune surveillance.

## Evidence for an IgG4 Bias in Some Tumors

IgG4 is usually the least-represented IgG subclass in human serum, comprising less than 4 % of the total IgG, but high IgG4 levels can occur in particular conditions, usually following repeated or chronic exposure to an antigen. Elevated levels of IgG4 in tissues and in serum are associated with inflammation in a range of chronic pathological conditions, such as rheumatoid arthritis [35], IgG4-related diseases (IgG4-RD) [36], and pemphigus vulgaris [37]. Elevated levels of IgG4 are also associated with immune tolerance under conditions of chronic exposure to a specific antigen, such as tolerance to bee venom in beekeepers [38•] or reduced allergic symptoms after allergen-specific immunotherapy in atopic individuals [14, 39].

IgG4 responses have also been reported in different cancers such as melanoma [40, 41••, 42••], extrahepatic cholangiocarcinoma [43, 44], pancreatic cancer [45], and glioblastoma [46••].

Tumor lesions are pathological conditions which present features resembling chronic inflammation. Some of these features may be (a) formation of tertiary lymphoid structures, shown to contain functional germinal centers [47•] where antigen-driven antibody responses may occur [48, 49] and (b) infiltration of M2-type macrophages and Foxp3^+^ regulatory T cells (Treg cells). IgG4 antibodies and IgG4^+^ B cells were detected together with Tregs in the tumor microenvironment of pancreatic cancer, cholangiocarcinoma [50], and melanoma lesions [41••, 51]. These tumor types were also characterized by Th2-biased environments with local expression of IL-10, IL-4, VEGF [41••], and TGF-β [50], mediators known to trigger B cells to produce IgG4 (Fig. 1b). IgG4 was found to positively correlate with Tregs and to negatively correlate with cytotoxic T lymphocytes [50], supporting its involvement of immune tolerance in cancer. Furthermore, ex vivo studies suggested that melanoma and B cell cross-talk can trigger elevated expression of IL-10 and VEGF, inducing B cells to produce IgG4 [41••]. Several carcinomas and cancer cell lines are found to produce IL-10 and FoxP3 [52], indicating that tumors may promote a biased Th2 response which supports IgG4, re-educating host immune responses and escaping the immune clearance. Consistent with this, elevated serum IgG4 levels have been associated with poorer prognosis in biliary tract cancers [43] and in malignant melanoma [41••, 42••].

Whether any tumor-associated IgG4 antibodies are tumor reactive is still unclear, although early evidence suggests this may be true in malignant melanoma [41••] and glioblastoma [46••]. Supernatants from ex vivo cultured B cells, isolated from melanoma patient blood and lesions, showed reactivity against melanoma cells in a cell-based ELISA [53], even if further analysis will be required to assess the specificity for tumor-associated antigens and exclude an allo-reactivity. Furthermore, tumor-exosome-reactive IgG4 and IgG2 antibodies were found in sera from glioblastoma patients [46••]. In other diseases, such as rheumatoid arthritis, IgG antibodies, including IgG4, were found to be specific for citrullinated fibrin and used as a serological marker [54]. On the other hand, in diseases such as IgG4-RD, IgG4 antibodies are usually found to be unspecific to disease-associated or auto-antigens [36]. Furthermore, only a small proportion of circulating IgG4 antibodies has been shown to recognize the specific allergen in atopic patients following allergen immunotherapy [55]. Thus, additional research would be required to ascertain the tumor reactivity and antigen specificity of tumor-associated IgG4 in patients with cancer.

## IgG4 Anti-inflammatory Properties: Blockade of IgG1-Mediated Effector Functions in Some Disease Settings Including Cancer

Evidence in different disease settings points to the ability of IgG4 to impair the effector functions of other immunoglobulins such as IgG1. In an in vivo model of myasthenia gravis, it was shown that IgG4 prevented IgG1-mediated internalization and degradation of acetylcholine receptors (AchRs) [25]. In human melanoma xenograft models in mice, partly reconstituted with human immune effector cells, tumor antigen-specific IgG4 could inhibit IgG1-mediated restriction of subcutaneous tumor growth [41••].

IgG4 could exert its inhibitory functions binding through the Fab (tumor-specific IgG4) or binding through the Fc (unspecific IgG4). The potential mechanisms by which IgG4 can interfere with the immune activating functions of IgG1 and of other class antibodies such as IgE are discussed below.

### IgG4 Competes with IgG1 for the Binding to the Antigen but Has Poor Ability to Trigger Effector Functions

The affinity of IgG4 for FcγRI is of the same order of magnitude as that of IgG1, while its affinities for FcγRIIa and FcγRIIIa are much lower than those of IgG1. FcγRs expressed on immune effector cells are engaged by antibodies to trigger antibody-dependent cellular phagocytosis (ADCP) and antibody-dependent cellular cytotoxicity (ADCC). FcγRIIa expressed on macrophages and phagocytes is involved in ADCP [56], while FcγRIIIa is expressed on NK cells and plays a crucial role in ADCC [57]. For this reason, even if IgG4 is able to bind FcγRI, the poor affinity to the other activating receptors may result in lower potency to mediate ADCC [58–61], and ADCP compared to IgG1. In general, IgG4 effector function could depend on the relative expression of FcγRs on the effector cells. Furthermore, unlike IgG1, IgG4 is not able to trigger complement-dependent cytotoxicity (CDC) [59].

In a scenario where IgG4 competes with IgG1 for the binding to the antigen, the poor effector function of IgG4 could result in the blocking of the potential IgG1-mediated effect (Fig. 1b).

There are mainly two mechanisms by which IgG4 could compete with IgG1 for the binding to the tumor. Firstly, according to CSR temporal model, IgG4 antibodies developed in the lesion should have higher affinity for the target antigen compared with IgG1, therefore competing for the binding to the tumor cells. The second is based on the theory that FAE is a mechanism that could, in certain conditions, increase IgG4 avidity to the antigen. Bi-specific IgG4 antibodies, able to simultaneously bind to the same or related antigens on target cells, are unlikely if Fab-arm exchange occurs in the blood stream with other IgG4 antibodies of unrelated specificity [25]. However, this might occur if clonally related antibodies against the same antigen or antibodies against different antigens on the same target cells co-localize at a site of inflammation [32], such as in tumor microenvironments.

### High-affinity IgG4 Can Take Part in IgG1 IC Co-engaging FcγRIIb with Activating FcγRs

Another possible mechanism of action of IgG4 could be mediated through the binding to the inhibitory receptor FcγRIIb. There is evidence that IgG4 can bind FcγRIIb with higher affinity than other IgG subclasses (Table 1) [12]. FcγRIIb is known to play a crucial role in regulating both innate (macrophage, mast cell, and basophil activation) and adaptive immunity (DC activation and antigen cross-presentation). It is also involved in B cell and plasma cell fate during the immune response [62]. Since FcγRIIb is able to exert its inhibitory action only if co-engaged with activating FcγRs [25], it has been speculated that high-affinity IgG4 could form ICs together with other antibodies, such as IgG1, and co-engage both FcγRIIb and activating FcγRs, thus dampening FcγR-mediated processes (Fig. 1b). According to this hypothesis, IgG4 might be able to exert an inhibitory effect even if its concentration is lower than IgG1 [32].

### IgG4 Can Bind IgG1 via Fc-Fc Interactions

IgG4 has been shown to bind other IgGs, in particular IgG1, via Fc-Fc interactions [63, 64••]. Two crystal structures of IgG4-Fc show two interfaces for Fc-Fc interactions localized in IgG4 C_H_2 and C_H_3 domains. Residues from these two interfaces belong to a consensus binding site for Fc-binding proteins such as neonatal Fc receptor (FcRn), TRIM21 [65], rheumatoid factor, staphylococcal protein A, streptococcal protein G, and the Herpes simplex virus type 1 (HSV-1) gE-gI receptor [66], and some residues also belong to aggregation-prone motifs [67]. The Fc-Fc interactions observed in the IgG4-Fc crystal structures may provide a model for the tendency of IgG4 to aggregate [24••]. Indeed, a study of therapeutic monoclonal antibodies showed that IgG4 has a higher tendency to aggregate than IgG1 [68].

A rheumatoid factor-like activity of IgG4 was observed first in rheumatoid arthritis [69, 70] and recently in autoimmune pancreatitis [71]. IgG4-Fc and not Fab was found to bind the heavy chain of all IgG1 subclasses [71]. Furthermore, Fc-Fc interaction was demonstrated between IgG4 and conformationally altered IgG4 or IgG1 immobilized on a solid-phase [63]. It was furthermore shown that IgG4 Fc-Fc interactions require partial dissociation of the IgG heavy chains [64••]. The authors hypothesized that IgG4 could act as a scavenger, by binding to IgG fragments and preventing their unfolding and formation of aggregates [64••].

Tumor microenvironments are rich in proteases such as matrix metalloproteases (MMP), which support extracellular matrix degradation, tissue remodeling, and promotion of cancer cell metastasis. An additional effect of these enzymes may be to partly dissociate or cleave host-produced or therapeutic antibodies [72]. An association between an inflammatory environment and the cleavage of autoantibodies was also found in rheumatoid arthritis [73]. In vitro studies showed that microbial origin and tumor-related proteases are also able to cleave IgG1 in a conserved region at the lower hinge/C_H_2 interface. Full cleavage to F(ab′)_2_ fragments is preceded by an intermediate with one intact heavy chain [74]. The same authors reported cleaved antibodies in breast cancer tumor extracts and suggested that cleaved IgG antibodies are likely present in tumor microenvironments. The cleaved antibodies have impaired FcR binding and effector functions but comparable antigen-binding capacity to that of intact antibodies. These damaged antibodies could therefore compete for the binding to the antigen and block the functional effects of undamaged IgGs [74], suggesting that proteolytic activity in tumor microenvironments could impair IgGs as a possible mechanism of tumor escape from host immune surveillance [72, 74].

In the proteolytic conditions of a tumor microenvironment, with cleaved IgG1 antibodies bound to tumor antigens, the cleavage of one of the heavy chains at the junction between the lower hinge and the C_H_2 domain may cause a partial dissociation of the cleaved heavy chain from the rest of the antibody. The partly dissociated IgG1 could facilitate the binding of IgG4-Fc resulting in the blockade, via Fc-Fc interactions, of single-chain cleaved IgG1 on the surface of tumor cells (Fig. 1b). This would probably require a high local concentration of IgG4 to overcome the affinity of the IgG1-Fc region for Fcγ receptors. However, the concentration of immunoglobulins in tumors is currently unknown. Fc-Fc mediated interactions could potentially interfere with IgG1-mediated effector functions or result in intracellular uptake and clearance of the target from the tumor cell surface.

## Implications of Employing IgG4 Subclass Antibodies in Cancer Immunotherapy

### Passive Immunotherapy

IgG4, together with IgG2, is the preferential subclass in the design of therapeutic antibodies, when the recruitment of the immune cells is undesired (receptor-blocking antibodies) or unnecessary (payload delivery antibodies) [75]. Therapeutic IgG4s can undergo FAE with endogenous IgG4. Depending on the mechanism of action of each specific therapeutic antibody, hinge stabilization (S228P mutation) could be a design consideration to prevent FAE in vivo [26].

IgG4 is generally considered an anti-inflammatory antibody due to its functionally monovalent properties, its poor ability to bind C1q and the activating low-affinity FcγRs. Nevertheless, IgG4 binds FcγRI with a K_A_ of the same order of magnitude as IgG1 and IgG3, and the inhibitory FcγRIIb receptor with higher affinity than the other subclasses [12, 76]. Thus in principle, it may be able to trigger effector functions. To eliminate any residual effector functions, therapeutic IgG4 antibodies can be engineered with mutations (such as L235E) that abrogate FcγR interactions [77, 78].

The overall anti-inflammatory activity of IgG4s, despite their affinity for FcγRI, could be related to their functional monovalency [15]. Therapeutic IgG4s carrying the S228P mutation are monospecific due to their inability to undergo FAE and would be able to crosslink antigens and to form small or large ICs. Large IgG4-ICs are able to bind the activating low-affinity FcγRs [79]. For this reason, during the design of IgG4 therapeutic antibodies, it may be important to incorporate both mutations that stabilize the hinge and those that completely abrogate Fcγ receptor binding.

### Active Immunotherapy

Triggering a patient response against cancer may be a desirable feature of active cancer immunotherapy. The emerging field known as AllergoOncology includes the use of IgE antibodies and antibody responses in cancer therapy [80]. IgE class switching may occur in a relatively naïve IgM-secreting B cell (direct route) or in an isotype-switched IgG4-expressing B cell (indirect route) [81]. In a clinical context, where the tumor lesion is infiltrated with IgG4^+^ B cells expressing high-affinity tumor antigen-specific IgG4s [41••], a valid tool for active immunotherapy could be the implementation of immunization approaches that support tumor-localized isotype switching from IgG4 to more potent IgE antibodies, via the indirect route. The result may be in situ production, of tumor antigen-specific IgE with high affinity for tumor targets that could mediate potent ADCC and ADCP against tumor cells and restrict tumor progression. Alternative approaches such as treatment with engineered IgE antibodies against cancer antigens may activate effector cells by signaling through IgE Fc receptors, as a means of bypassing IgG4-mediated Fc blockade mechanisms. In the last decade, mounting evidence supports the idea of engineering IgE antibodies and vaccination approaches that result in IgE-biased immune responses against cancer as powerful tools for cancer immunotherapy [82–85].

## Conclusion

The structural and functional attributes of IgG4 described here render it unique among the IgG subclasses. Its ability to undergo FAE may be only partly responsible for its low affinity for activating receptors expressed on immune effector cells and consequently low capacity to engage these cells to trigger ADCP and ADCC and also for its poor ability to bind C1q and fix complement. IgG4 is considered an antibody with low immunoactivatory properties, and it is a suitable subclass for the design of therapeutic antibodies when effector functions are not desirable. However, a better understanding of its properties and functions in vivo is still required to inform the design and optimization of IgG4-based therapeutic antibodies. Reports of IgG4 antibodies and IgG4+ B cells in different cancers suggest the involvement of IgG4 in tumor escape from immune surveillance through a number of potential mechanisms, including IgG4 blockade of IgG1-mediated effector functions. However, IgG4 and its roles in cancer inflammation remain unclear. Dissecting the cross-talk between cancer and humoral immunity and the conditions that can promote IgG4-biased “regulatory” responses can inform the design of novel therapeutic antibodies with improved immune activatory and effector functions and reduced susceptibility to tumor-associated immune blockade.

## Abbreviations

Ig: Immunoglobulin
V_L_: Light chain variable domain
V_H_: Heavy chain variable domain
CDRs: Complementarity determining regions
C_H_: Constant domain
Fab: Fragment antigen binding
Fc: Fragment crystallizable
HL: Heavy chain/light chain half molecule
CSR: Class switch recombination
SHM: Somatic hypermutation
AID: Activation-induced cytidine deaminase
IL: Interleukin
VEGF: Vascular endothelial growth factor
FcγR: Fcγ receptor
FAE: Fab-arm exchange
CDC: Complement dependent cytotoxicity
ADCC: Antibody-dependent cellular cytotoxicity
ADCP: Antibody-dependent cellular phagocytosis
